# Absorbable Artificial Dura Versus Nonabsorbable Artificial Dura in Decompressive Craniectomy for Severe Traumatic Brain Injury: A Retrospective Cohort Study in Two Centers

**DOI:** 10.3389/fsurg.2022.877038

**Published:** 2022-07-01

**Authors:** Zhong-Ding Zhang, Li-Yan Zhao, Yi-Ru Liu, Jing-Yu Zhang, Shang-Hui Xie, Yan-Qi Lin, Zhuo-Ning Tang, Huang-Yi Fang, Yue Yang, Shi-Ze Li, Jian-Xi Liu, Han-Song Sheng

**Affiliations:** ^1^ Department of Neurosurgery, The Second Affiliated Hospital and Yuying Children’s Hospital of Wenzhou Medical University, Wenzhou, China; ^2^ The Second School of Medicine, Wenzhou Medical University, Wenzhou, China; ^3^ West China School of Public Health, Sichuan University, Chengdu, China; ^4^ Department of Neurosurgery, Yueqing Affiliated Hospital of Wenzhou Medical University, Wenzhou, China

**Keywords:** artificial dura, TBI - traumatic brain injury, decompressive craniectomy, transcalvarial cerebral herniation, duraplasty

## Abstract

**Background:**

Severe traumatic brain injury (TBI) patients usually need decompressive craniectomy (DC) to decrease intracranial pressure. Duraplasty is an important step in DC with various dura substitute choices. This study aims to compare absorbable dura with nonabsorbable dura in duraplasty for severe TBI patients.

**Methods:**

One hundred and three severe TBI patients who underwent DC and dura repair were included in this study. Thirty-nine cases used absorbable artificial dura (DuraMax) and 64 cases used nonabsorbable artificial dura (NormalGEN). Postoperative complications, mortality and Karnofsky Performance Scale (KPS) score in one year were compared in both groups.

**Results:**

Absorbable dura group had higher complication rates in transcalvarial cerebral herniation (TCH) (43.59% in absorbable dura group vs. 17.19% in nonabsorbable dura group, *P* = 0.003) and CSF leakage (15.38% in absorbable dura group vs. 1.56% in nonabsorbable dura group, *P* = 0.021). But severity of TCH described with hernial distance and herniation volume demonstrated no difference in both groups. There was no statistically significant difference in rates of postoperative intracranial infection, hematoma progression, secondary operation, hydrocephalus, subdural hygroma and seizure in both groups. KPS score in absorbable dura group (37.95 ± 28.58) was statistically higher than nonabsorbable dura group (49.05 ± 24.85) in one year after operation (*P* = 0.040), while no difference was found in the rate of functional independence (KPS ≥ 70). Besides, among all patients in this study, TCH patients had a higher mortality rate (*P* = 0.008), lower KPS scores (*P* < 0.001) and lower functionally independent rate (*P* = 0.049) in one year after surgery than patients without TCH.

**Conclusions:**

In terms of artificial biological dura, nonabsorbable dura is superior to absorbable dura in treatment of severe TBI patients with DC. Suturable nonabsorbable dura has fewer complications of TCH and CFS leakage, and manifest lower mortality and better prognosis. Postoperative TCH is an important complication in severe TBI which usually leads to a poor prognosis.

## Introduction

Traumatic brain injury (TBI) is a leading cause of death and disability which can affect people of all ages (1). Its incidence is increasing in developing countries for inadequate traffic laws and imperfect construction safety regulations, which brings a big challenge for hospitals and society (2). TBI can be simply divided into mild, moderate and severe based on Glasgow Coma Scale (GCS). Moderate and severe TBI patients are often accompanied with mixed brain injuries (such as contusions and hemorrhages) and need neurosurgical treatment (3).

Decompressive craniectomy (DC) is the traditional surgical procedure for TBI patients to relieve severely raised intracranial pressure (ICP) by removing part of the skull (4). With the great progress of materials and technology, artificial dura mater was widely used to repair dura defects in DC for TBI patients. Most of the recent medical dura products are made from biological materials or polymer materials, and can be divided into absorbable and nonabsorbable dura based on biodegradability (5–7). Previous researches usually concentrated on a single material, and reported quite different surgical outcomes in complications and neurofunctional recovery (8–10). However, there is still a lack of high-quality study which horizontally compared different kinds of dura under the same conditions and give practical advice. The advantages, disadvantages and limitations of absorbable and nonabsorbable dura are still unclear.

In this study, we used absorbable dura made from bovine tendon type I collagen or nonabsorbable dura made by porcine pericardium for duraplasty in severe TBI patients to compare their complications and prognosis after DC.

## Method

This study is based on the medical records and follow-up data of severe TBI patients from The Second Affiliated Hospital of Wenzhou Medical University and Yueqing Affiliated Hospital of Wenzhou Medical University. Patients were divided into absorbable group and nonabsorbable group according to the kind of dura used in DC. Demographic characteristics and injuries condition were compared to confirm the comparability of the two groups. Follow-up was conducted for at least one year. Postoperative complications, mortality and Karnofsky Performance Scale (KPS) score were compared in two groups. Additional analysis of TCH was performed. The study was approved by the Ethics Committees of The Second Affiliated Hospital and Yuying Children's Hospital of Wenzhou Medical University. Written informed consent was obtained.

### Patients

The consecutive cohort includes 103 patients with severe TBI (GCS ≤ 8) who received decompressive craniectomy and dura repairment in The Second Affiliated Hospital of Wenzhou Medical University and Yueqing Affiliated Hospital of Wenzhou Medical University (Yueqing People’s Hospital) from January 2017 to December 2020. The two hospitals are both tertiary medical center and affiliated hospital of Wenzhou Medical University. Both hospitals have regional trauma centers and provide emergency neurosurgical care for TBI.

Here are the include and exclude criteria of this study: Include criteria: (1). The clear history of TBI and patients’ GCS is between 3 and 8. (2). Decompressive craniectomy is primary and only on one site. (3). Patients received artificial dura repairment. Exclude criteria: (1). Posterior cranial fossa trauma (2). Patients only with simple epidural hematoma. (3). History of intracranial surgery (4). Serious underlying diseases may influence surgical prognosis, including coagulopathy, heart failure, malignant tumor, etc. (5). Clinical, radiological, or follow-up data is incomplete.

**Figure 1:**
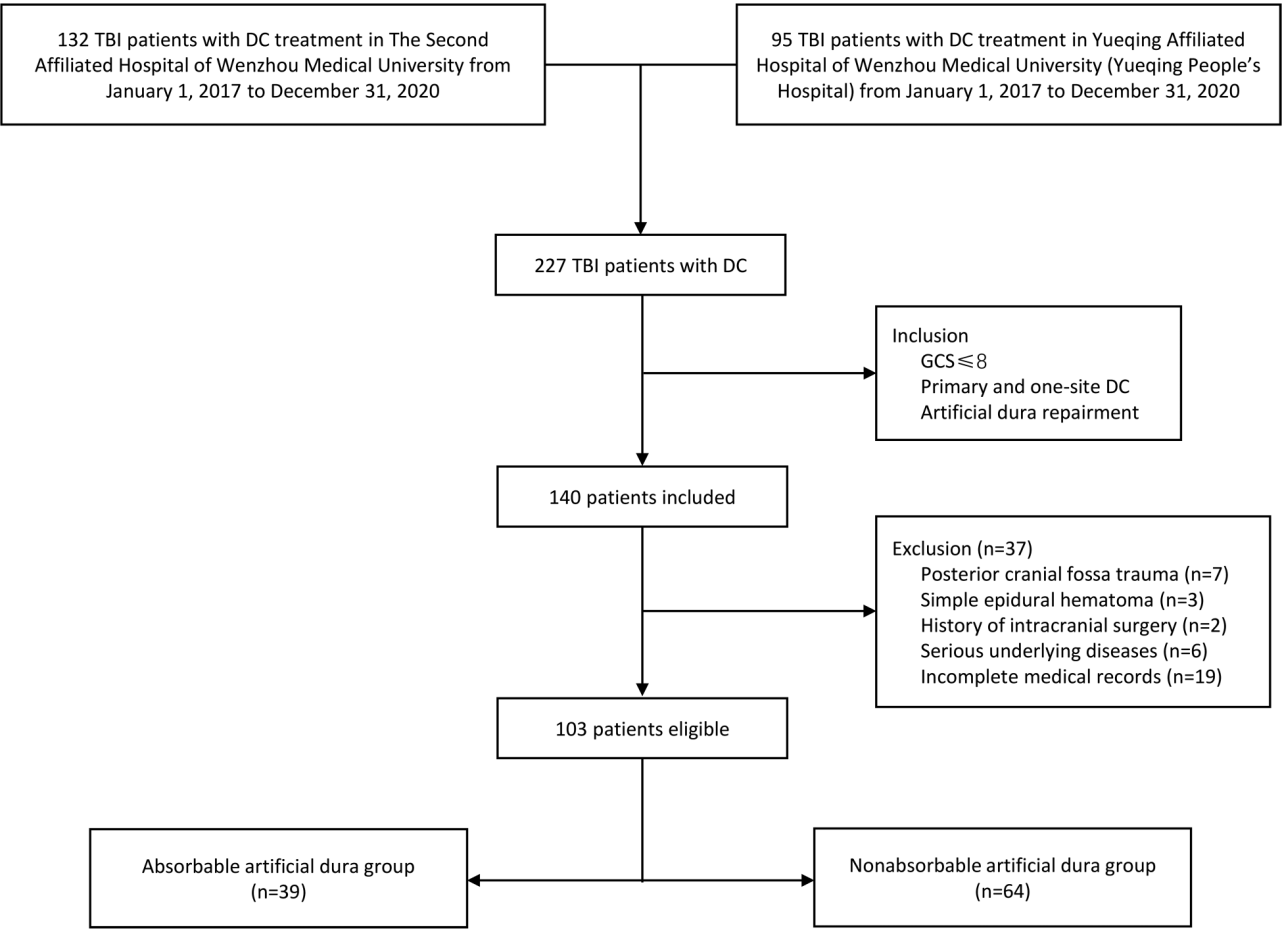
Flowchart of study.

Finally, we sorted out 103 severe TBI patients who meet the criteria from 227 hospitalized TBI patients with DC treatment in two centers. Patients were divided into two groups according to which kind of dura was used. Thirty-nine cases used absorbable artificial dura while 64 cases used nonabsorbable artificial dura (see Figure 1).

### Dural Material

Absorbable dura is “DuraMax” manufactured by Tianxinfu Medical Appliance Co., Ltd (Beijing, China), which is made of high pure bovine tendon type I collagen and processed into biological membrane scaffold. The product is sterilized by ethylene oxide. Nonabsorbable dura is “NormalGEN” manufactured by Guanhao Biotech Co., Ltd (Guangzhou, China). The product is made of porcine pericardium tissue by cross-linking treatment and is sterilized by γ-ray irradiation. Absorbable dura is sutureless while nonabsorbable dura is suturable.

### Indication of DC

The use of DC is controversial and there is still no consensus on its indications. In our centers, we performed DC on severe TBI patients in the following situation: (1). Clinical symptoms of brain herniation such as dilated pupils; CT showed manifestations of the hernia (obvious midline shift, cisterna ambiens compression) with traumatic lesions of cerebral contusion, hemorrhage, edema or ischemia; (2). Severe TBI patients with ICP increased over 30 mmHg in 30 min; (3). Acute TBI patients with progressive disturbance of consciousness; CT showed obvious mass effects of traumatic lesions; and first-line treatment such as osmotic therapy and hypothermia therapy cannot control the increasing ICP.

However, DC is generally not performed on dying patient suffering from long-time brain hernia with the symptoms of bilateral dilated pupil fixation, loss of light response, respiratory arrest, blood pressure instability, etc.

### Surgical Procedure

In two centers, the operation would be carried out immediately when the indication of DC is clear and informed consent is obtained. After patients were generally anesthetized and intubated, operations were performed with close monitoring of vital signs including heart rate, blood pressure and respiratory rate. The dura mater was cut in radial or arched shape and suspended to the skull window. The deficient area would be fixed with artificial dura. In the Absorbable Dura group, the dura was trimmed and then pasted to the brain parenchyma, making sure that the edge of artificial dura has covered the primary dura; In the Nonabsorbable Dura group, the artificial dura was trimmed and then tension-reduced sutured to the remaining dura mater. Finally, we placed the drain between the scalp and dura mater and then suture the scalp. A layer of gelatin sponge was placed between the dura mater and the drain to prevent the dura dislodgement in both groups. Patients with excessive bleeding or low blood volume will receive blood transfusion. After the surgery, patients were transferred to intensive care unit with careful observation of disease development.

### Data Collection

**Figure 2:**
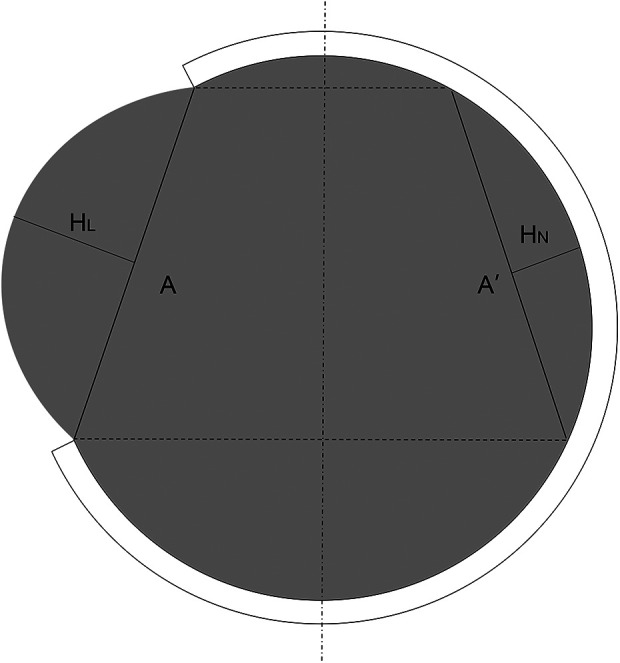
Measurement of herniation volume on maximal hernial layer of CT (A: diameter of craniectomy; H_L_: maximum perpendicular distance from hernial brain tissue to line A on lesion side. H_N_: maximum perpendicular distance from normal brain tissue to symmetrical line A’ on non-lesion sides).

Clinical data were collected from the electronic medical record system of the hospital. Midline shift was measured as the maximum perpendicular distance between the septum pellucidum and midline on the plane of interventricular foramen on the preoperative CT imaging. The diameter of craniectomy was measured as the attachment line of the inner plate of the skull on postoperative CT imaging. Transcalvarial cerebral herniation (TCH) is defined as “brain tissue displacement is above the surface of the outer skull plate over one centimeter.” We used “maximum hernial distance” and “herniation volume” to describe and compare the severity of TCH. We reviewed the CT image in 14 days postoperatively to confirm if postoperative TCH happened. If so, we measured the maximum distance from the outer plate of the skull to obtain hernial distance and used the formula put forward by Liao et al. to calculate the herniation volume (11) (see Formula (1) and Figure 2).


(1) $$V=\frac{1}{2}A^{2}\Delta h=\frac{1}{2}A^{2}\left(H_{L}-H_{N}\right)$$

**Figure 3:**
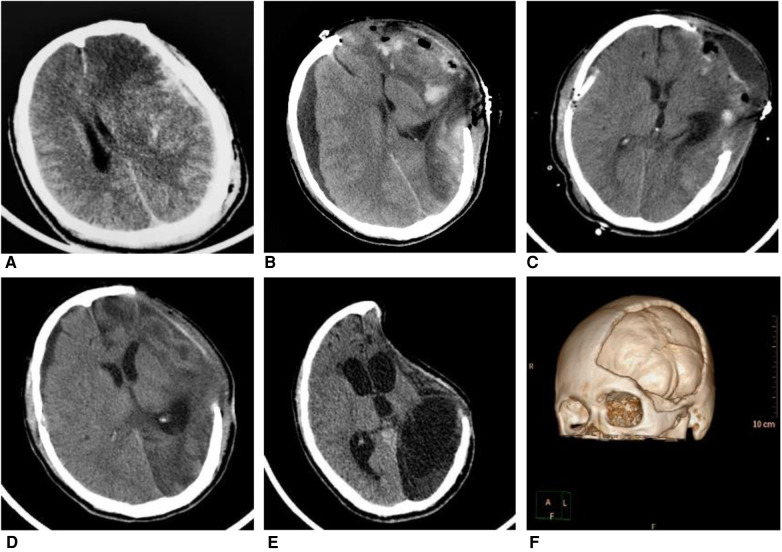
A 31-year-old man fell from a height of three meters after electric shock, whose GCS is 4 points at admission. Patient received decompressive craniectomy (DC) and absorbable dura repairment. (**A**) Computed tomography (CT) image at admission showed an acute subdural hematoma on the left side. (**B**) CT at 11 days after DC showed subdural hygroma on the right side and herniation of brain tissue on the left side. (**C**) Patient received burr hole irrigation and drainage 13 days after DC, postoperative CT showed the reduction of subdural hygroma. (**D**) CT at one month after DC showed shrinkage of left-sided brain tissue (**E**) Six months after DC, CT indicated the syndrome of the trephined (**F**) 3D reconstruction of the CT scan of this patient after DC.

Secondary operation was an emergency operation required for a worsening condition after primary DC, which did not include cranioplasty. Follow-up was conducted one year after the decompressive craniectomy, and we use KPS to access the functional recovery of patients (12). KPS ≥ 70 is the standard of “functionally independent.”

### Statistical Analysis

SPSS 25.0 (IBM Corp., Armonk NY, USA) software was used for data analysis. Variables between the absorbable dura group and nonabsorbable dura group were compared with students’ t test. The differences in rates were tested by chi-square test, continuous correction chi-square test or Fisher exact tests, if appropriate. The data is regarded as statistically significant while *P* < 0.05

## Result

There are totally 103 patients included in this study. All patients were diagnosed as severe TBI (GCS ≤ 8) and received standard decompressive craniectomy with artificial dura repairment. Patients were divided into two groups according to the type of artificial dura mater: 39 cases used absorbable (sutureless) dura while 64 cases used nonabsorbable (suturable) dura. There was no statistical significance between two groups in terms of age, sex, causes, GCS at admission. Preoperative CT was reviewed and no statistical significance was found in imaging features between two groups. See Table 1 for details.

**Table 1:** Baseline information in absorbable dura group and nonabsorbable dura group.

Factors	Absorbable Dura group (*n* = 39)	Nonabsorbable Dura group (*n* = 64)	t /χ2 value	*P* value
Age	50.53 ± 15.69	47.25 ± 19.44	0.891	0.375
Sex			1.006	0.316
Male	32(82.05%)	47(73.44%)		
Female	7(17.95%)	17(26.56%)		
Causes			0.826	0.719
Traffic accidents	20(51.28%)	27(42.19%)		
Falls	16(41.03%)	31(48.44%)		
Others	3(7.69%)	6(9.38%)		
GCS Score	5.95 ± 1.62	6.22 ± 1.41	−0.891	0.375
Midline Shift	7.73 ± 6.66	7.45 ± 6.47	0.190	0.850
Preoperative CT diagnoses				
Subdural hematoma	34(87.18%)	51(79.69%)	0.943	0.331
Epidural hematoma	9(23.08%)	18(28.13%)	0.319	0.572
Subarachnoid hemorrhage	36(92.31%)	58(90.63%)	0.000	1.000
Intracerebral hematoma	2(5.13%)	5(7.81%)	0.015	0.903
Contusion	33(84.62%)	56(87.50%)	0.172	0.679
Pneumocrania	11(28.21%)	13(20.31%)	0.845	0.358
Injury-surgery interval (h)	7.33 ± 5.88	7.63 ± 5.40	−0.257	0.798
Diameter of craniectomy	87.90 ± 12.92	85.76 ± 15.28	0.680	0.499

*GCS, Glasgow Coma Scale.*

Surgical outcomes indicated that absorbable dura group had a higher complication rate than nonabsorbable dura group in postoperative TCH (43.59% in absorbable dura group vs. 17.19% in nonabsorbable dura group, *P* = 0.003) and incisional CSF leakage (15.38% in absorbable dura group vs. 1.56% in nonabsorbable dura group, *P* = 0.021). However, two groups had no difference in intracranial infection, hydrocephalus, subdural hygroma and seizure (see Table 2). There was also no statistical difference in rates of hematoma progression and secondary operation. KPS score in absorbable dura group (37.95 ± 28.58) was lower than nonabsorbable dura group (49.05 ± 24.85) in one year after operation (*P* = 0.040), while functional independence was similar between both groups. Mortality in one year did not reach statistical difference though that of absorbable dura group was higher. Besides, we tried to compare the severity of TCH between two groups, but no statistical difference was found on the part of hernial distance and herniation volume, though the herniation volume in absorbable dura group was slightly bigger (see Table 3). However, we found that patients with postoperative TCH had a higher mortality rate (*P* = 0.008), lower KPS scores (*P* < 0.001) and fewer functionally independent patients (*P* = 0.049) in one year after surgery than patients without postoperative TCH. The rate of secondary operation in TCH patients was also higher, though not statistically (see Table 4).

**Table 2:** Surgical outcomes of absorbable dura group and nonabsorbable dura group.

Prognosis	Absorbable Dura group (*n* = 39)	Nonabsorbable Dura group (*n* = 64)	t /χ2 value	*P* value
TCH	17(43.59%)	11(17.19%)	9.086	**0** **.** **003**
Intracranial infection	2(5.13%)	1(1.56%)	0.193	0.660
Hydrocephalus	9(23.08%)	15(23.43%)	0.002	0.967
Subdural hygroma	10(25.64%)	19(29.69%)	0.196	0.658
Incisional CSF leak	6(15.38%)	1(1.56%)	5.290	**0**.**021**
Seizure	2(5.13%)	1(1.56%)	0.193	0.660
Progress of hematoma	12(30.77%)	19(29.69%)	0.013	0.908
Secondary operation	11(28.21%)	15(23.44%)	0.292	0.589
Death	9(23.08%)	7(10.94%)	2.722	0.099
KPS score	37.95 ± 28.58	49.05 ± 24.85	−2.076	**0**.**040**
Functionally independent	9(23.1%)	18(28.1%)	0.319	0.572

*TCH, Transcalvarial Cerebral Herniation; CSF, Cerebrospinal Fluid; KPS, Karnofsky Performance Scale*.

**Table 3:** TCH severity comparison between absorbable dura group and nonabsorbable dura group.

	Absorbable Dura group (*n* = 17)	Nonabsorbable Dura group (*n* = 10)	*t* value	*P* value
Hernial distance (mm)	14.55 ± 4.31	14.59 ± 5.10	−0.023	0.982
Herniation volume (ml)	62.56 ± 22.09	56.13 ± 40.94	0.458	0.655

**Table 4:** Prognosis between patients with TCH and without TCH.

Prognosis	Patients with TCH (*n* = 27)	Patients without TCH (*n* = 76)	t /χ2 value	*P* value
Secondary operation	9(33.33%)	16(21.05%)	1.635	0.201
Death	9(33.33%)	7(9.21%)	7.093	**0** **.** **008**
KPS score	29.26 ± 27.02	50.38 ± 24.51	3.744	**<0**.**001**
Functionally independent	3(11.11%)	23(30.26%)	3.872	**0**.**049**

*TCH, Transcalvarial Cerebral Herniation; KPS, Karnofsky Performance Scale*.

## Discussion

TBI is a high-incidence disease with multiple and complicated pathophysiology. Severe TBI, defined as GCS ≤8 after resuscitation, within 48 h of injury, usually leads to high mortality and high disability (13). Treatment of increased ICP is central to TBI patients and it is reported that maximal medical therapy is ineffective on about 10%–15% of severe TBI patients with increased ICP (14). DC is a surgical procedure to relieve severely increased ICP by removing a part of skull and making room for swollen brain tissue, which is lifesaving to severe TBI patients. However, researches also show that DC can bring some potential complications and increase the rate of vegetative state and severe disability (4, 15, 16). Therefore, it’s important to figure out the risk factors during DC to improve the prognosis of patients.

As far as the 1930s, surgeons have realized the importance of dura repairment in TBI and use heteroplastic or autoplastic dural grafts for repairment (17). With the development of industry and technology, various artificial dura substitutes were invented and applied in surgery. At present, the main application of dural replacement materials can be divided into four types: autologous grafts, allografts, xenografts and synthetic grafts. Xenografts were preferred in DC with the evacuation of acute subdural hematomas for its advantage of good biocompatibility and mechanical properties (18–20). However, different biologic dura grafts were made from different biomaterials and were processed differently, which resulted in imparity in tensile strength, biodegradability and biocompatibility.

Biodegradability is an important parameter of artificial dura, which is also the major discrepancy between DuraMax and NormalGEN we used in the biologic character. Absorbable materials can be gradually replaced by regenerated dural tissue during the degradation process while nonabsorbable materials complete or partial remain in the cranial cavity permanently. Which type of dura is better remains controversial. In early practice, materials scientists and neurosurgeons usually used synthetic materials. Absorbable materials such as polyglactin 910, polydioxanone and polytetrafluoroethylene (EPTFE) usually manifested better histomorphology outcomes and less sequelae than xenografts and other synthetic nonabsorbable dura (18, 21). However, after the 2000s, several biological dural substitutes gradually replaced previous synthetic materials because of their better biocompatibility. Absorbable collagen-based dura was proved to be safer than former products (22). And nonabsorbable dura deriving from bovine, equine or porcine pericardium also exhibits good outcomes in animals and patients in recent researches (8, 23, 24).

It is reported that DuraMax can be degraded and absorbed within three months while NormalGEN has not been absorbed over one year (25). These two products were widely used in neurosurgery for duraplasty in the last decade in China. According to the existing reports, they were both proved to have good biocompatibility and positive surgical outcomes (8, 25, 26). In our study, we concentrate on their prognosis differences in DC for severe TBI patients and discuss which type of dura is more suitable in this situation.

In our research, the nonabsorbable dura NormalGEN group presented with fewer postoperative complications including incisional CSF leakage and TCH, and better long-term neurofunction recovery outcomes than absorbable dura DuraMax group. It’s comprehensible that the rapid degradation of dura in absorbable dura group may cause defect and lead to higher rates of CSF leakage. Similarly, Neulen et al. (27) found that the nonabsorbable dura made of bovine pericardium show less adhesion to brain tissue and better CSF tightness than absorbable dura made from bovine tendon type I collagen in pig model, indicating that pericardium materials might be more suitable in TBI patients. Besides, the difference of usage in surgery (NormalGEN is sutureless while Nonabsorbable dura is suturable) also resulted airtightness disparity. Moreover, CSF dynamics disturbances after DC is also an important cause of CFS leak, and artificial dura can reduce it by maintaining tension. Zerris et al. (28) found that the mechanical properties of nonabsorbable bovine-pericardium-made dura were similar to native dura while absorbable tendon-type-I-collagen-made dura is too fragile to quantify, which could be another possible reason. Even so, there are many researches that indicate sutureless absorbable dura can reduce CSF leakage compared to autologous grafts (25, 29, 30). However, a recent randomized controlled clinical trial showed that rapid closure DC which only covered the exposed brain parenchyma with surgical exhibited no difference on CSF leak and other complications compared to DC with water-tight duraplasty (31). The result of this trial is controversial for its small sample and non-statistical-test design (32). Most neurosurgeons believe that artificial dura with a higher suture strength and better sealing performance should be firstly considered in patients with high risk of CSF leakage (33).

We also compared our results with similar products reported in previous literature. DuraGen (Integra Lifesciences, Plainsboro, NJ, USA), a sutureless and absorbable dural substitute graft composed of bovine tendon type I collagen, is similar to DuraMax we used. It is reported in humans that the CSF leak rate of DuraGen varied from 0.4% to –8.3% in various kinds of neurosurgeries, and the infectious complications rate varied from 0.9% to 4.8% (34–36). However, we failed to find reports of DuraGen which concentrate on TBI. The higher CFS leak rate (15.38%) of DuraMax in our study might attribute to the alteration of CSF circulation after DC, or product differences. Nonabsorbable dura made from porcine, bovine or equine pericardium is also widely used in duraplasty, and there are some products similar to NormalGEN, such as DuraGuard (Biovascular Corp., Minneapolis, MN, USA), PeriGuard (Synovis, St. Paul, Minnesota, USA) and Heart®, (Bioteck, Vicenza, Italy). Pericardium-made products have shown good outcomes in neurosurgeries of tumor resection, Chiari I malformation decompression, microvascular decompression and DC (24, 37, 38). Sun et al. used bovine pericardium membranes in DC for severe TBI, and reported 5.64% of infection, 5.13% of CSF leak and 3.08% of seizure in 195 patients (8), which are generally similar to our outcomes of NormalGEN.

DC creates space for the brain to expand through skull defect, which can reduce ICP and increase cerebral perfusion pressure. However, severe incision hernia, which is usually called TCH or external cerebral herniation, is a harmful postoperative complication of DC. After the skull was removed, the only mechanical barriers to prevent swelling brain tissue are artificial dura and soft tissue, so the expansion of brain is unconstrained (39). TCHs in DC is related to the brain edema caused by ischemia reperfusion and changes of microcirculation, which gives rise to the block of capillaries (40). Obstruction of blood flow leads to further bulge and damage of brain tissue, aggravates the inflammatory response, and even causes necrosis of the neurocyte (41). A wide DC (not less than 12 × 15 or 15 cm diameter) is recommended for its lower mortality and better neurologic outcomes (4). Hinge decompressive craniectomy (hDC) might avoid this complication but it has not been popularized yet (42).

There is no diagnose criterion of TCH after DC from any guideline, we reviewed several previous studies and chose “brain tissue displacement is above the surface of the outer skull plate over 1 cm” as the standard according to our clinical experience and for the convenience of judgment on CT images (11, 41, 43). Recently, Liao et al. (11) have proposed a simplified formula to measure the volume of brain tissue herniated through the craniectomy. And we used it to calculate and compare the severity of TCH between two groups. We found that patients used absorbable dura are more likely to have TCH 14 days after surgery. But hernial extent and volume of TCH patients with absorbable dura were not significantly different compared to those with nonabsorbable dura may be because of the small sample size. We consider that the attachment method and extensibility of dura are the major factors contributing to this difference. The nonabsorbable dura NormalGEN is tightly sutured to the original dura and can bear more strain to prevent TCH. We also compare the prognosis of patients with TCH and without TCH. The mortality rate and KPS scores in one year in TCH patients are statically significantly poorer, and we believe that the different rate of TCH in the two groups is the considerable factor leading to the different endings. In our clinical observation, most TCH reduced and disappeared after positive ICP decreasing treatment while extremely severe ones usually died.

TCH is the early complication of DC while subdural hygroma and hydrocephalus are late complications (44). A recent study shows that TCH and subdural hygroma can be risk factors of posttraumatic hydrocephalus after DC (43). As previously mentioned, TCH can lead to ischemic insults and axonal secondary injury, which can cause ventricular dilation and variation of CSF circulation. Some patients without cranioplasty even had the syndrome of the trephined in a few months (see Figure 3). However, we didn’t find an incidence difference of subdural hygroma or hydrocephalus between both groups though there are more TCH occurring in absorbable dura group. The result may due to the higher early mortality of TCH patients in absorbable dura group and the small sample size of our data. How the biodegradability of dura influences the circulation of cerebrospinal fluid remains to be a mystery. The pathology and clinical value of TCH after DC still need more convincing evidence.

The surgical duration of nonabsorbable artificial dura group is slightly longer because dura stitches spend time. Some extremely severe patients may be more inclined to use absorbable dura for its sutureless operation and shorter surgical duration. However, we also noticed that the case number of nonabsorbable dura group is larger than that of the absorbable dura group, because some experienced surgeons may prefer to use the former for its better airtightness. Since the two devices are both domestic and affordable to most of the family, economic factor was out of consideration in this study.

According to our clinical data, we consider that severe TBI patients are more likely to benefit from biological suturable nonabsorbable dura with good airtightness and elasticity for dura repair in DC. However, sutureless absorbable dura also has its advantages of small patches application in some locations difficult to reach, shorter surgery duration and better histomorphologic outcomes (45–47). A recent study reported a new absorbable dura made of Poly L Lactic Acid which shows good mechanical properties and represents better anti-adhesive property than NormalGEN in preclinical studies (48). Artificial dura made by novel synthetic substitutes is still the important aspect of future research. All in all, different types of artificial dura may have their own merits in various neurosurgical applications, and more clinical evaluation and comparison should be done to figure out their optimum adaptation and provide ideas for the development of new products.

## Conclusion

Nonabsorbable biological dura is superior to absorbable biological dura in the treatment of severe TBI patients with DC. Suturable nonabsorbable dura manifests lower rates of postoperative TCH and CFS leakage, and leads to a better prognosis. Besides, it is worth mentioning that patients with postoperative TCH are usually related to higher mortality and poorer neurofunctional rehabilitation.

## Limitation

The study was retrospective. Absorbable dura is sutureless while nonabsorbable dura is suturable, so the selection of dura and patient might not be random sometimes. The outcomes of this study may be affected by neurosurgeons’ surgical skill and personal preference of dura products. Besides, the low utilization rate of the ICP monitor also prevented us from elaborating on the mechanism of TCH. The convinced conclusion needs randomized controlled trials and large multi-center researches with a bigger sample. Besides, comparisons of more dura substitutes should be done to figure out the role of dura in TCH after DC.

## Data Availability

The raw data supporting the conclusions of this article will be made available by the authors, without undue reservation.
